# Integrating network pharmacology, transcriptomics, and experimental validation: Compound Baixianpi Formula targets IL-17A to inhibit dual PI3K-AKT/JAK2-STAT3 pathways for psoriasis improvement

**DOI:** 10.1186/s13020-026-01386-0

**Published:** 2026-05-22

**Authors:** Lin Tang, Yanli Wang, Yue Zhou, Chunli Gan, Jinhui Wang

**Affiliations:** 1 Department of Medicinal Chemistry and Natural Medicine Chemistry, College of Pharmacy, Harbin Medical University, 157 Health Road, Nangang District, Harbin City, 150081 Heilongjiang Province China; 2 College of Pharmaceutical Engineering and Biotechnology, Zhejiang Pharmaceutical University, No. 888, East Section of Yinxian Avenue, Yinzhou District, Ningbo City, Zhejiang Province China

**Keywords:** Traditional Chinese Medicine, FFBXP, Psoriasis, Network pharmacology, Transcriptomics, IL-17A, PI3K-AKT/JAK2-STAT3 signaling

## Abstract

**Background:**

Psoriasis is an immune-mediated chronic inflammatory skin disease. Existing therapies have limitations, necessitating the development of new treatment approaches. Compound Baixianpi Formula (FFBXP) is a clinically effective topical Chinese herbal formula, but its material basis and mechanism of action remain unclear.

**Materials and methods:**

FFBXP components were identified using UHPLC-Q-Orbitrap HRMS. Core targets and pathways were screened by integrating network pharmacology and transcriptomics analyses. The effects of FFBXP on skin lesions, histopathology, oxidative stress, and inflammatory mediators (IL-17A, IL-23, TNF-α) were evaluated using a IMQ mouse psoriasis model and a TNF-α-stimulated HaCaT cell model. Key mechanisms were validated through molecular docking, qRT-PCR, Western blot, and immunofluorescence techniques.

**Results:**

Forty-two active components were identified in FFBXP. In vivo experiments demonstrated that FFBXP significantly improved erythema and skin lesion infiltration in psoriatic mice, while reducing levels of inflammatory cytokines (IL-17A, IL-23, TNF-α) and oxidative stress markers (MDA). In vitro experiments confirmed that FFBXP dose-dependently inhibited TNF-α-induced proliferation in HaCaT cells, reduced inflammatory cytokine levels, mitigated oxidative stress, and promoted apoptosis. Network pharmacology and transcriptomics analysis indicated its mechanism involves IL-17, PI3K-AKT, and JAK2-STAT3 signaling pathways. Molecular docking showed that six core active ingredients (berberine, resveratrol, quercetin, catechin, kaempferol, and osthol) had good binding activity with key target proteins. Further mechanism validation revealed FFBXP significantly decrease IL-17A expression and inhibited phosphorylation of downstream PI3K, AKT, JAK2, and STAT3 proteins.

**Conclusion:**

This study employs a combined strategy of network pharmacology, transcriptomics, and experimental validation to elucidate for the first time that FFBXP exerts its anti-psoriasis effects by targeting IL-17A and collaboratively inhibiting two key signaling pathways: PI3K-AKT and JAK2-STAT3.

**Supplementary Information:**

The online version contains supplementary material available at 10.1186/s13020-026-01386-0.

## Introduction

Psoriasis is a clinically common, refractory chronic inflammatory skin disease, recognized by the World Health Organization (WHO) as one of the top ten intractable diseases. Globally, it affects approximately 125 million people, with a prevalence rate of 1–3%, and its incidence shows a gradual upward trend [1]. Clinically, psoriasis typically characterized by localized or widespread scaly erythema or plaques, often covered with silvery-white scales, and is accompanied by symptoms such as skin itching, dryness, cracking, and even bleeding [2, 3]. The etiology and pathogenesis of psoriasis are complex, and modern medicine generally attributes it to the combined effects of genetic factors and internal/external environmental influences [4]. Key drivers of its development include immune systemic imbalance., abnormal immune cell activity, excessive proliferation of keratinocytes, and increased production of inflammatory factors such as IL-23 and IL-17A [5]. Currently, clinical treatments for psoriasis primarily focus on symptom control to improve patients' quality of life, including pharmacological agents, phototherapy, and combination approaches. However, many medications are associated with adverse effects such as liver and kidney impairment, gastrointestinal discomfort, and skin dryness [6]. In recent years, biologics have been widely adopted for psoriasis treatment, but their application is limited due to strict eligibility criteria and high costs.

IL-17A, the primary cytokine secreted by Th17 cells, drives psoriatic inflammation by prompting keratinocytes to release pro-inflammatory factors (IL-6, IL-8, TNF-α) [7] and chemokines. Clinical studies have shown significantly increased infiltration of Th17 cells and IL-17A expression in psoriatic lesions, with serum IL-17A levels positively correlating with disease severity (assessed by the PASI score) [8]. Accumulating evidence confirms that targeting IL-17A or its receptor effectively blocks key pathogenic pathways in psoriasis [9]. Currently, IL-17 inhibitors such as secukinumab and ixekizumab have become important therapeutic options in China: their excellent efficacy and favorable safety profile have markedly improved patient outcomes [10]. Dysregulated PI3K-AKT signaling contributes to psoriasis progression by promoting keratinocyte excessive proliferation and impairing their differentiation, and enhancing Th17 cell responses [11]. IL-17A and other inflammatory cytokines (IL-23, TNF-α) further activate this pathway, forming a vicious cycle that not only induces keratinocyte dysfunction but also regulates Th17 cell differentiation [12]. Pharmacological inhibition of PI3K-AKT signaling-for instance, with the PI3K inhibitor LY294002 [13] or natural compounds like resveratrol [14–16] reduces inflammatory responses and abnormal cell proliferation, underscoring the pathway’s therapeutic potential. The JAK-STAT signaling pathway plays a critical role in psoriasis pathogenesis by regulating inflammatory responses and cell proliferation. Its abnormal activation by various cytokines disrupts immune cell function and keratinocyte homeostasis [17]. Key cytokines (IL-6, IL-23, IL-17A) specifically activate the JAK1/2-STAT3 axis, which sustains disease progression [18–20].

Traditional Chinese Medicine (TCM) has shown remarkable effectiveness in treating psoriasis, offering comprehensive benefits including minimal side effects, reduced relapse rates, and improved quality of life [21]. In TCM theory, psoriasis involves intertwined heat, toxin, dampness, stasis, and wind [22, 23]. Compound Baixianpi Formula (FFBXP) is a topical Chinese herbal preparation developed through long-term clinical practice for the treatment of inflammatory skin conditions. It consists of nine herbs: *Dictamnus dasycarpus* Turcz (Bai Xian Pi, BXP), *Cnidium monnieri* (L.) Cusson (She Chuang Zi, SCZ), *Spatholobus suberectus* Dunn (Ji Xue Teng, JXT), *Tribulus terrestris* L. (Ji Li, JL), *Smilax glabra* Roxb*.* (Tu Fu Ling, TFL), *Reynoutria japonica Houtt.* (Hu Zhang, HZ), *Styphnolobium japonicum* (L.) Schott (Huai Hua, HH), *Sophora flavescens* Aiton (Ku Shen, KS), and *Pseudolarix amabilis* (J.Nelson) Rehder (Jin Qian Song, JQS). According to TCM compatibility principles, BXP serves as the monarch herb for clearing heat and detoxifying; SCZ, KS, and TFL act as minister herbs to enhance dampness-drying and itch-relieving effects; JXT, HZ, and HH function as assistant herbs to promote blood circulation and resolve plaques; JL and JQS serve as envoy herbs to guide the formula to the skin surface. Collectively, FFBXP exerts heat-clearing, detoxifying, wind-dispelling, dampness-drying, blood-circulating, and itch-relieving effects. Research has shown that key components of FFBXP exhibit distinct anti-psoriatic mechanisms: BXP contains dictamnine, which alleviates skin inflammation by modulating IL-23 and suppressing p-P38/p-ERK1/2 signaling [24]. SCZ inhibits HaCaT cell proliferation and shows clinical efficacy in reducing psoriatic plaques [25, 26]. Other herbs like JXT and TFL work through multiple pathways including NK cell activation and keratinocyte suppression [27, 28]. Notably, HZ formulations demonstrate hormone ointment-comparable efficacy with better safety [29], while HH-based treatments show reliable clinical results [30]. Despite these advantages, the complex nature of herbal compounds and unidentified active components continue to challenge mechanistic understanding, highlighting the need for further research.

Recent advancements in analytical technologies have enabled more in-depth investigations: liquid chromatography-mass spectrometry (LC–MS) allows for high-resolution characterization of herbal components, while network pharmacology elucidates multi-target mechanisms through systematic drug-target-disease network analysis. Transcriptomics further reveals genome-wide gene expression changes following TCM treatment, uncovering molecular-level therapeutic mechanisms. These innovative approaches collectively facilitate comprehensive exploration of herbal remedies like Dictamnus dasycarpus in psoriasis management, bridging traditional knowledge with modern scientific validation [31].

This study used UHPLC-Q-Orbitrap HRMS to analyze FFBXP components, and evaluated its therapeutic effects in an imiquimod-induced mouse psoriasis model and a TNF-α-stimulated HaCaT cells model. By integrating network pharmacology prediction and transcriptome analysis, it was found that FFBXP may exert therapeutic effects by regulating the PI3K-AKT and JAK2-STAT3 signaling networks through IL-17A, and its multi-t target mechanism was validated through protein experiments.

## Materials and methods

### Main reagents

FFBXP was provided by Ningbo Medicinal Materials Co., Ltd. HaCaT cells were purchased from Shanghai Fuheng Biotechnology Co., Ltd. Imiquimod Cream was purchased from Henan Topfond Pharmaceutical Co., Ltd. Benvitimod Cream was purchased from Guangdong Zhonghao Pharmaceutical Co., Ltd. The CCK-8 Cell Counting Kit and qRT-PCR kit were purchased from Nanjing Vazyme Biotech Co., Ltd. The GSH, SOD, and MDA assay kit were purchased from Nanjing Jiancheng Bioengineering Institute. Mouse IL-17A ELISA Quantification Kits, Mouse IL-23 ELISA quantitative detection kit and Mouse TNF-α ELISA detection kit were purchased from Shanghai Enzyme Linked Biotechnology Co., Ltd. The antibodies (GAPDH, IL-17A, p-PI3K, PI3K, p-AKT, AKT1, p-JAK2, JAK2, p-STAT3, STAT3) used in the experiment were obtained from Affinity Biosciences. The chemicals (Osthole, Resveratrol, Quercetin and Kaempferol) used in the experiment were obtained from Yuanye Biosciences.

The proteins (AKT1, IL17A, JAK2 and PIK3CA) used in the experiment were obtained from Hangjing Biosciences.

### Chemical composition analysis of FFBXP

Sample Preparation: Take 200 μL of FFBXP medicine, add 600 μL of methanol, extract by ultrasound for 30 min, centrifuge at 4 ℃ and 12000 rpm for 10 min, and take 100 μL of supernatant for analysis. Chromatographic Conditions: Using Thermo Vanquish Flex UHPLC system and Waters ACQUITY UPLC HSS T3 chromatographic column (2.1 × 100 mm, 1.8 μm). Mobile phase: Phase A (0.1% formic acid aqueous solution), Phase B (acetonitrile); Flow rate of 0.3 mL/min; Column temperature 40 ℃; The injection volume is 6.0 μL. Gradient Elution Program: 0–1 min (2% B), 1–14 min (2–30% B), 14–25 min (30–100% B), 25–28 min (100% B), 28–28.1 min (100–2% B), 28.1–30 min (2% B). Mass Spectrometry Conditions: Using Thermo Q Exactive^™^ Mass spectrometer, ESI ion source. Parameter settings: ion source voltage 3.7 kV (+)/3.5 kV (−); Capillary temperature 320 ℃; Sheath gas pressure 30 psi, auxiliary gas pressure 10 psi; Evaporation temperature 300 ℃; The collision gas (N2) pressure is 1.5 mTorr. Data Acquisition: Full scan resolution of 70000, scanning range of m/z 100–1500; DD-MS2 resolution 17500, collision energy 10/30/60 V. Data Analysis: Use Progenesis QI 3.0 software for data processing, identify compounds based on retention time, precise mass number (error < 5 ppm), secondary mass spectrometry fragments, and other information, combined with reference standards and theoretical databases.

### Preparation of FFBXP

The herbal formula comprises the following components: *Dictamnus dasycarpus* Turcz*.* (Bai Xian Pi, BXP) 5 g, *Cnidium monnieri* (L.) Cusson (She Chuang Zi, SCZ) 3 g, *Spatholobus suberectus* Dunn (Ji Xue Teng, JXT) 9 g, *Tribulus terrestris* L. (Ji Li, JL) 6 g, *Smilax glabra* Roxb*.* (Tu Fu Ling, TFL) 15 g, *Reynoutria japonica Houtt.* (Hu Zhang, HZ) 9 g, *Styphnolobium japonicum* (L.) Schott (Huai Hua, HH) 5 g, *Sophora flavescens* Aiton*.* (Ku Shen, KS) 4.5 g, and *Pseudolarix amabilis* (J.Nelson) Rehder (Jin Qian Song, JQS) 15 g, and we have verified the plant names in EXD against the Plant List database (http://mpns.kew.org). For preparation, the herbs were weighed according to the specified proportions and sealed in a double-layered decoction bag. A tenfold volume of water (relative to the total herb weight) was added, and the mixture was decocted for 40 min. The resulting decoction was filtered through gauze to harvest the liquid extract. The herbs were then subjected to a second decoction with additional water, boiled for 15 min, and filtered again. The two filtrates were combined and concentrated to achieve a final crude drug concentration of approximately 0.9295 g/mL (Convert the adult equivalent dose to a mouse dose of 9.295 g/kg, and set it as the low-dose group dosage. Administer at a volume of 0.2 mL/20 g, and calculate the mass concentration of the FFBXP medicinal solution as 0.9295 g/mL).

### Construction of psoriasis mouse model

Sixty male BALB/c mice (6 weeks old) were acclimatized for 1 week, then divided into control (n = 10) and model (n = 50) groups. Mice were purchased from Hangzhou Ziyuan Experimental Animal Technology Co., Ltd (SCXK (Zhe) 2024-0004). The study was approved by the Ethics Committee of the School of Pharmacy, Harbin Medical University (Ethics Number: IRB3029725). Psoriasis-like lesions were induced in model mice by daily 5% imiquimod application (62.5 mg/day) for 7 days. Successful modeling was confirmed by persistent erythema, edema, and scaling. Modeled mice were further divided into five treatment groups (n = 10): control, Benvitimod (0.096 g/day), and FFBXP-H (3.718 g/mL), FFBXP-M (1.859 g/mL), and FFBXP-L (0.9295 g/mL) doses via 10-min daily wet compresses (0.2 mL) for 7 days. Control and model groups received saline. After treatment, some skin was fixed in paraformaldehyde for paraffin sectioning, some in glutaraldehyde, and the rest was frozen at −80 °C. Serum and tissue were stored at −80 °C for future analysis.

### PASI score of mouse skin

During the administration period, the changes in erythema, scales, and lesion infiltration of each group of mice were recorded daily, and PASI scores were given based on the degree of erythema, scales, and infiltration. The PASI scoring criteria were: 0 for asymptomatic, 1 for mild symptoms, 2 for moderate symptoms, 3 for severe symptoms, and 4 for extremely severe symptoms. The total score was the sum of the three scores, and the detailed scoring criteria are shown in Supplementary materials 1.

### Hematoxylin & Eosin and Masson staining

Mouse skin tissues were harvested and fixed in paraformaldehyde. After fixation, tissues were rinsed under running water, dehydrated with a graded ethanol series, cleared in xylene, embedded in paraffin wax and sectioned at 3 μm thickness using a microtome. Subsequently, sections were processed according to standard protocols, with H&E staining and Masson’s trichrome staining to evaluate skin histopathological changes and collagen deposition, respectively.

### Network pharmacological analysis

The 2D structures and SMIES structures of 42 components of FFBXP were collected from Pubchem (https://pubchem.ncbi.nlm.nih.gov/) database. The corresponding targets were retrieved from Swiss Target Prediction database. GeneCards (https://www.genecards.org/) were used psoriasis target retrieval. The overlapping genes of 42 components and psoriasis were applied to build a network of a protein–protein interaction (PPI). Finally, core genes were subjected to GO and KEGG pathway analyses, and the binding energies of the components and their targets were calculated after visualization with Autodock and PyMOL.

### Transcriptomics analysis

Total RNA was extracted from mouse skin tissues using the TRIzol method, and RNA concentration and purity were assessed via a Nanodrop spectrophotometer (A260/A280 ratio). RNA integrity was evaluated using Agilent 4150 Bioanalyzer (RNA Integrity Number, RIN ≥ 7.0). Only qualified samples were used for subsequent database construction. Paired-end (PE) libraries were constructed according to the mRNA seq Lib Prep Kit manual, and after passing the Agilent 4150 quality inspection, perform 150 bp double ended sequencing using Illumina Novaseq 6000/MGISEQ-T7 platform. Raw sequencing data quality was evaluated using FastQC, Trimomatic to remove adapters and filter low-quality reads (Q ≤ 25, base ratio > 60% or N content > 5%) to obtain clean reads. Using HISAT2 (http://daehwankimlab.github.io/hisat2/) to align clean reads to the reference genome, Obtain mapped reads. Use featureCounts to count gene read counts and calculate FPKM (http://subread.sourceforge.net/) values for gene expression level standardization. Differential analysis was conducted based on DESeq2 (| log2FC |> 1 and Padj < 0.05), and Venn maps of three differentially expressed genes, Control/Model/FBBXP, were drawn using Jvenn. Perform GO functional and KEGG pathway enrichment analysis using clusterProfiler with statistical significance defined as P < 0.05 to reveal the biological functions and pathway characteristics of differentially expressed genes.

### Molecular docking analysis

The crystal structure of IL-17A, PI3K, AK1, JAK2 and STAT3 protein was determined by the protein database (https://www.rcsb.org), and Dictamnine, Resveratrol, Kaempferol, Osthole, Quercetin and Catechin structures were obtained from the PubChem (https://pubchem.ncbi.nlm.nih.gov) database. Molecular docking simulations were performed through AutoDockTools-1.5.7 and visualized with PyMOL 2.5.2

### Surface plasmon resonance (SPR)

SPR-based binding assays were conducted on a Biacore T200 instrument (GE Healthcare) at 25 ℃, employing PBST (PBS containing 1% DMSO) as the buffer. Protein samples were dissolved in coupling buffer (10 μg/ml sodium acetate) and immobilised onto a CM5 chip. Protein immobilisation parameters were set as follows: pH 4.5, flow rate 20 μl/min, 25 ℃. Osthole, resveratrol, quercetin and kaempferol were diluted and injected at a flow rate of 10 μl/min for 120 s (contact phase), followed by 150 s (dissociation phase). Binding data were collected using the Biacore T200 evaluation software (GE Healthcare).

### HaCaTcells culture

Frozen HaCaT cells were thawed at 37 °C, suspended in complete medium, and centrifuged (1200 rpm, 3 min). After resuspension, cells were cultured at 37 °C with 5% CO₂. At 80–90% confluency, cells were trypsinized (0.25% trypsin–EDTA, 1 min), neutralized, centrifuged, and passaged at 1:2 ratio into fresh flasks under standard conditions.

### Cell proliferation

Log-phase HaCaT cells (5 × 10^4^ cells/mL) were seeded in 96-well plates (100 μL/well) and cultured for 12 h. After TNF-α stimulation (10 ng/mL, 24 h), cells were treated with FFBXP (0, 0.2, 0.4, 0.8, 1.2, 1.6, 2 mg/mL) for 48 h. Cell viability was assessed by CCK-8 assay (2 h incubation) measuring absorbance at 450 nm, with optimal FFBXP concentration selected for subsequent experiments.

### Flow cytometry detection of HaCaT cells apoptosis and ROS levels

After 48 h treatment, cells were trypsinized, washed, and resuspended in Binding Buffer. For apoptosis detection, cells were stained with Annexin V-FITC/PI (5 µL each, 10 min dark incubation) and analyzed by flow cytometry. For ROS measurement, cells were loaded with 10 µM DCFH-DA, washed, trypsinized, and resuspended in PBS before flow analysis within 1 h.

### ELISA kit for detecting IL-17A, IL-23, and TNF-α in serum

Serum and HaCaT cell samples were processed per ELISA kit protocols to quantify IL-17A, IL-23 and TNF-α levels, with concentrations determined from standard curves.

### Detection of SOD, GSH, and MAD in skin tissue kit

Skin tissues and HaCaT cell lysates were processed following manufacturer protocols for SOD, GSH, and MDA detection. Absorbance readings were measured and concentrations calculated using standard curves from each respective assay kit.

### Quantitative real-time polymerase chain reaction (qRT-PCR)

Total cellular RNA was extracted from cells using Trizol reagent (Magen, China) following the manufacturer’s standard protocol. Complementary DNA (cDNA) was synthesized via reverse transcription using HiScript IIQRT SuperMix for qRT-PCR. The mRNA levels of IL-17A were detected by quantitative real-time PCR with ChamQ SYBR qRT-PCR Master Mix. Primer sequences used are shown in Supplementary materials 2 and their specificity was confirmed using the BLAST algorithm of National Center for Biotechnology Information. Data were normalized to GAPDH and analyzed by 2^(− ΔΔCT)^ method.

### Western blot analysis

Total protein was extracted from skin samples and HaCaT cells using RIPA lysis buffer (Beyotime, China). Proteins were separated by SDS-PAGE and transferred to PVDF membranes. After blocking with 5% skim milk, membranes were incubated overnight at 4 °C with primary antibodies, followed by 1 h incubation with HRP-conjugated secondary antibodies at room temperature. Protein bands were visualized using ECL chemiluminescence and quantified with ImageJ software.

### Immunofluorescence

HaCaT cells were subjected to immunofluorescence staining for IL-17A. After rinsing with PBS, the cells were fixed with 4% formaldehyde for 30 min, permeabilized with 0.5% Triton X-100 for 10 min, and incubated with primary antibodies overnight at 4 °C and fluorescent secondary antibodies for 1 h in the dark. The cells were then stained with DAPI, treated with an anti-quenching sealant, and examined under a microscope.

### Statistical analysis

Data were dealt using the GraphPad Prism 9.0.0 software (GraphPad Software, La Jolla, CA). All data were represented as mean ± standard deviation (SD) and *p* < 0.05 was considered statistically different. Statistical analysis was performed by one-way analysis of variance (ANOVA) tests.

## Results

### FFBXP-main chemical composition analysis

**Fig. 1:**
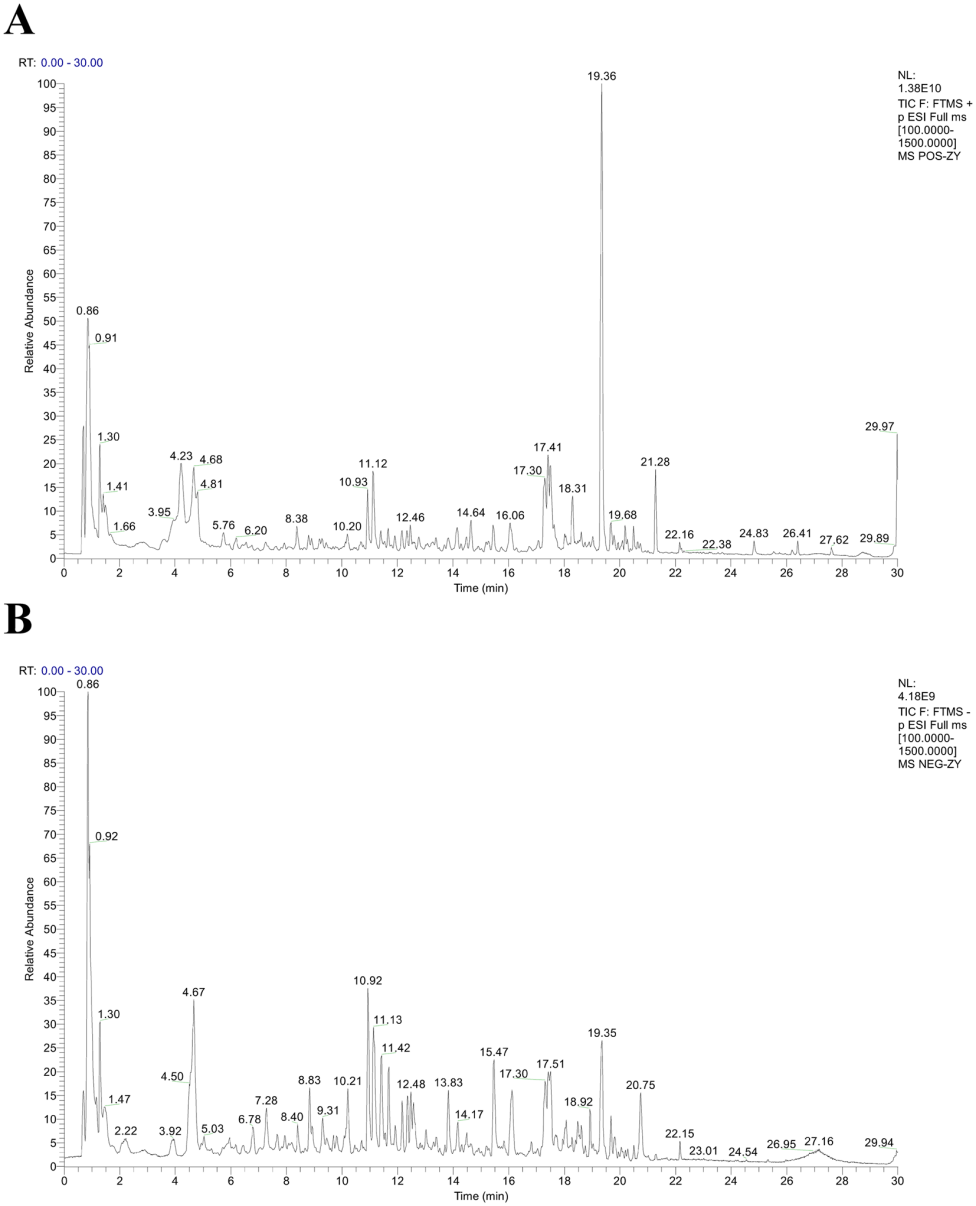
UHPLC-Q-Orbitrap HRMS chromatograms of FFBXP. **A** Total Ion Chromatography (TIC) of FFBXP detected in positive mode. **B** Total Ion Chromatography (TIC) of FFBXP detected in negative mode

UHPLC-Q-Orbitrap HRMS analysis identified FFBXP, 42 compounds included Cryptotanshinone, Emodin, Dictamnine, Liquiritigenin, Obacunone, Hyperoside, Resveratrol, Isoliquiritigenin, Chlorogenic acid, Baicalin, Quercetin, Naringenin et al. were identified or initially characterized. The ingredient name, retention time, mode, adducts, molecular formula, m/z, fragmentation score of FFBXP were listed and summarized in Table 1 and Fig. 1.

**Table 1:** 42 compounds in the FFBXP compositional identification

No	Compound	m/z	RT (min)	Mode	Adducts	Formula	Score
1	Osthole	245.1173	21.28	POS	M-e, M + H, 2 M + H	C_15_H_16_O_3_	98.50
2	Limonin	471.2018	18.93	POS	M + H, M + NH4	C_26_H_30_O_8_	94.38
3	Cryptotanshinone	297.1488	22.44	POS	M + H, M + Na	C_19_H_20_O_3_	93.88
4	Emodin	269.0452	20.75	POS-NEG	M−H	C_15_H_10_O_5_	91.75
5	Ononin	431.1340	14.29	POS	M + H	C_22_H_22_O_9_	90.88
6	Dictamnine	200.0706	18.31	POS	M + H	C_12_H_9_NO_2_	90.75
7	Liquiritigenin	255.0660	14.81	POS-NEG	M−H	C_15_H_12_O_4_	89.13
8	Biochanin a	283.0608	19.66	NEG	M−H	C_16_H_12_O_5_	88.38
9	Fraxinellone	233.1173	20.66	POS	M + H-2H2O, M + H-H2O, M + H	C_14_H_16_O_3_	87.50
10	Obacunone	455.2067	20.28	POS	M + H-H2O, M + H, M + NH4, M + Na, 2 M + H	C_26_H_30_O_7_	86.63
11	Physcion	285.0759	23.02	POS	M + H	C_16_H_12_O_5_	86.25
12	Bergapten	217.0495	18.20	POS	M + H	C_12_H_8_O_4_	85.88
13	Hyperoside	465.1030	10.68	POS	M + H	C_21_H_20_O_12_	85.13
14	Formononetin	269.0806	18.28	POS-NEG	M + H, M + Na	C_16_H_12_O_4_	84.63
15	Astragalin	449.1079	12.16	POS	M + H	C_21_H_20_O_11_	83.13
16	Methoxsalen	217.0494	17.33	POS	M + H	C_12_H_8_O_4_	83.00
17	Resveratrol	227.0703	13.83	NEG	M−H, M + FA-H	C_14_H_12_O_3_	82.63
18	Emodin-8-beta-d-glucoside	431.0977	16.12	NEG	M-H	C_21_H_20_O_10_	81.13
19	Isorhamnetin	317.0653	17.54	POS	M + H	C_16_H_12_O_7_	80.50
20	Genistein	271.0600	16.82	POS-NEG	M + H	C_15_H_10_O_5_	79.88
21	Isoliquiritigenin	255.0658	17.95	POS-NEG	M−H	C_15_H_12_O_4_	79.38
22	Wogonoside	461.1084	16.37	POS	M + H	C_22_H_20_O_11_	79.25
23	Chlorogenic acid	353.0879	7.50	NEG	M−H	C_16_H_18_O_9_	78.88
24	Alloimperatorin	271.0964	19.93	POS-NEG	M + H	C_16_H_14_O_4_	77.63
25	Ferulic acid	177.0547	10.95	POS	M + H-H2O, M + H	C_10_H_10_O_4_	76.63
26	Isoquercitrin	465.1029	11.12	POS-NEG	M + H	C_21_H_20_O_12_	76.63
27	Daidzein	255.0653	14.66	POS-NEG	M + H	C_15_H_10_O_4_	76.25
28	Diosmetin	301.0703	17.45	POS-NEG	M + H	C_16_H_12_O_6_	75.88
29	Nicotiflorin	595.1660	11.76	POS	M + H	C_27_H_30_O_15_	75.75
30	Cynaroside	449.1080	11.65	POS	M + H	C_21_H_20_O_11_	75.38
31	Baicalin	447.0924	14.24	POS-NEG	M + H	C_21_H_18_O_11_	74.88
32	Sophoridine	249.1962	4.23	POS	M + H, M + Na, 2 M + H	C_15_H_24_N_2_O	74.13
33	Quercetin	303.0498	15.47	POS	M + H	C_15_H_10_O_7_	74.13
34	Naringenin	271.0611	16.81	NEG	M−H	C_15_H_12_O_5_	73.75
35	Astilbin	449.1087	11.42	POS-NEG	M−H, M + FA-H	C_21_H_22_O_11_	73.63
36	P-coumaric acid	147.0441	9.82	POS	M + H-H2O, M + H	C_9_H_8_O_3_	72.88
37	Calycosin	285.0757	15.74	POS-NEG	M + H, M + Na, M + K	C_16_H_12_O_5_	71.88
38	Rutin	611.1604	11.12	POS-NEG	M + H, M + Na, 2 M + H	C_27_H_30_O_16_	71.63
39	Catechin	289.0716	7.28	POS-NEG	M−H, M + FA-H	C_15_H_14_O_6_	71.50
40	Kaempferol	287.0548	17.20	POS	M + H	C_15_H_10_O_6_	70.13
41	Sophoricoside	433.1132	12.62	POS-NEG	M + H, M + Na	C_21_H_20_O_10_	68.25
42	5-hydroxymethylfurfural	127.0394	3.88	POS	M + H-H2O, M + H	C_6_H_6_O_3_	67.63

### Evaluation of efficacy of FFBXP in psoriasis mice

**Fig. 2:**
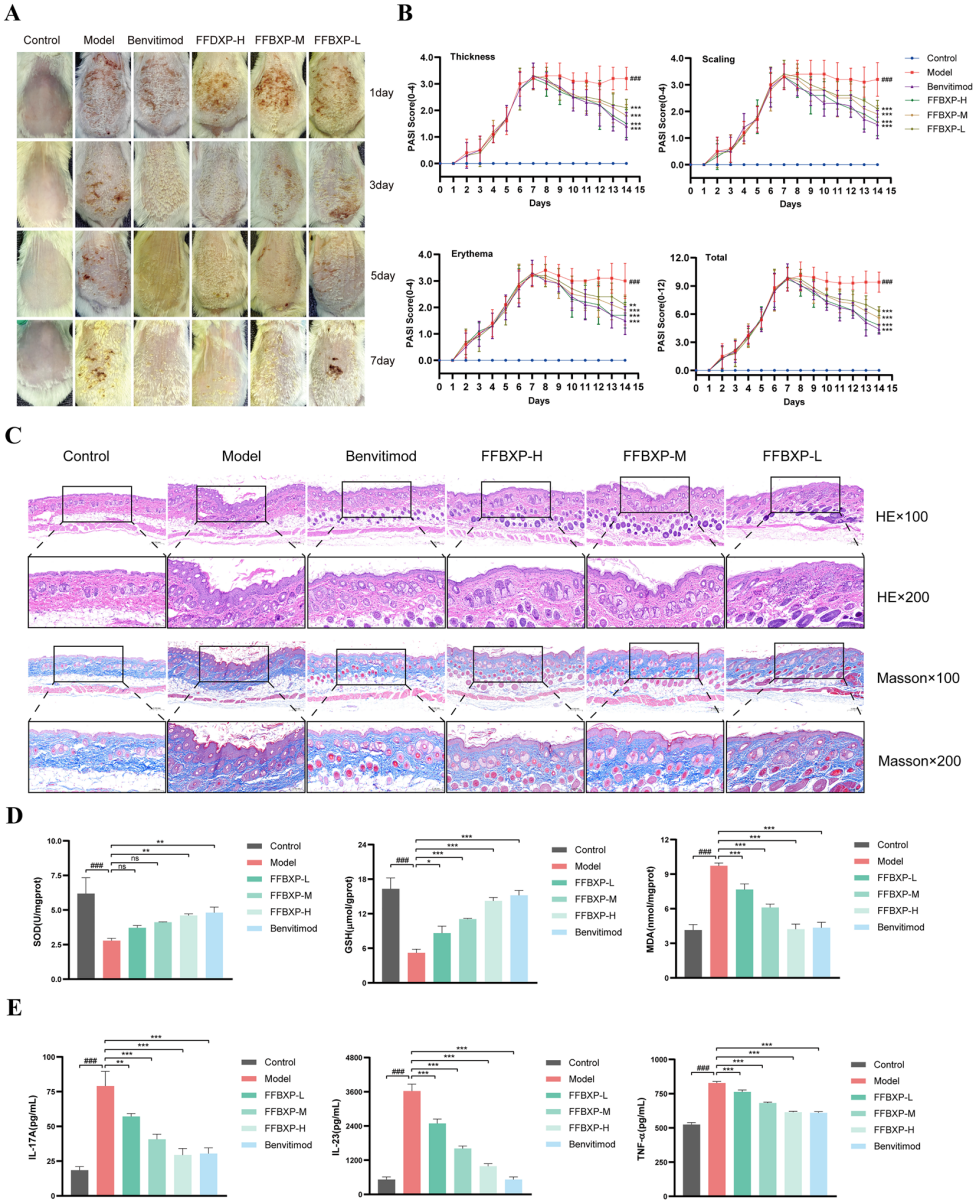
Evaluation of efficacy of FFBXP in psoriasis mice. **A** Skin images of mice in each group. **B** PASI score (plaque thickness, scale coverage area, erythema, PASI total score) (*n* = 10). **C** HE staining and Masson staining (*n* = 3). **D** Detection of oxidative stress factors SOD, GSH and MDA (*n* = 3). **E** Detection of inflammatory factors IL-17A, IL-23 and TNF-α (*n* = 3). ^###^*p* < 0.001 vs. Control; ^**^p < 0.01, ^***^p < 0.001 vs. Model

This study investigated the therapeutic effects of FFBXP in an imiquimod-induced psoriasis mouse model. The model group developed psoriasis-like lesions (thickened epidermis, scales, erythema), while FFBXP treatment (3–7 days) dose-dependently improved symptoms. By day 7, FFBXP-L showed mild erythema, FFBXP-M exhibited reduced scales and resolved erythema, and FFBXP-H achieved near-normal skin, matching Benvitimod's efficacy, confirming FFBXP’s potent therapeutic potential (Fig. 2A, B).

Histological analysis (Fig. 2C) showed the model group exhibited typical psoriatic pathological features, including epidermal hyperplasia, hyperkeratosis, and inflammatory cell infiltration, while FFBXP treatment dose-dependently reversed these histological abnormalities. FFBXP-L reduced hyperplasia, FFBXP-M showed mild hyperplasia, and FFBXP-H nearly normalized skin structure, comparable to Benvitimod, underscoring FFBXP’s efficacy in alleviating psoriatic skin damage. Quantitative analysis (Fig. S1) confirmed that FFBXP dose-dependently reduced epidermal thickness and inflammatory cell counts, consistent with the PASI scores and biochemical markers.

The study revealed psoriasis mice exhibited oxidative stress imbalance (reduced GSH/SOD, increased MDA) and elevated pro-inflammatory cytokines (IL-17A, IL-23, TNF-α). FFBXP treatment dose-dependently restored antioxidant defense systems and suppressed the production of inflammatory mediators, with high-dose FFBXP demonstrating comparable efficacy to Benvitimod in mitigating both oxidative damage and inflammation, highlighting its potential as a therapeutic agent for psoriasis (Fig. 2D, E).

### Network pharmacology analysis of FFBXP against psoriasis

**Fig. 3:**
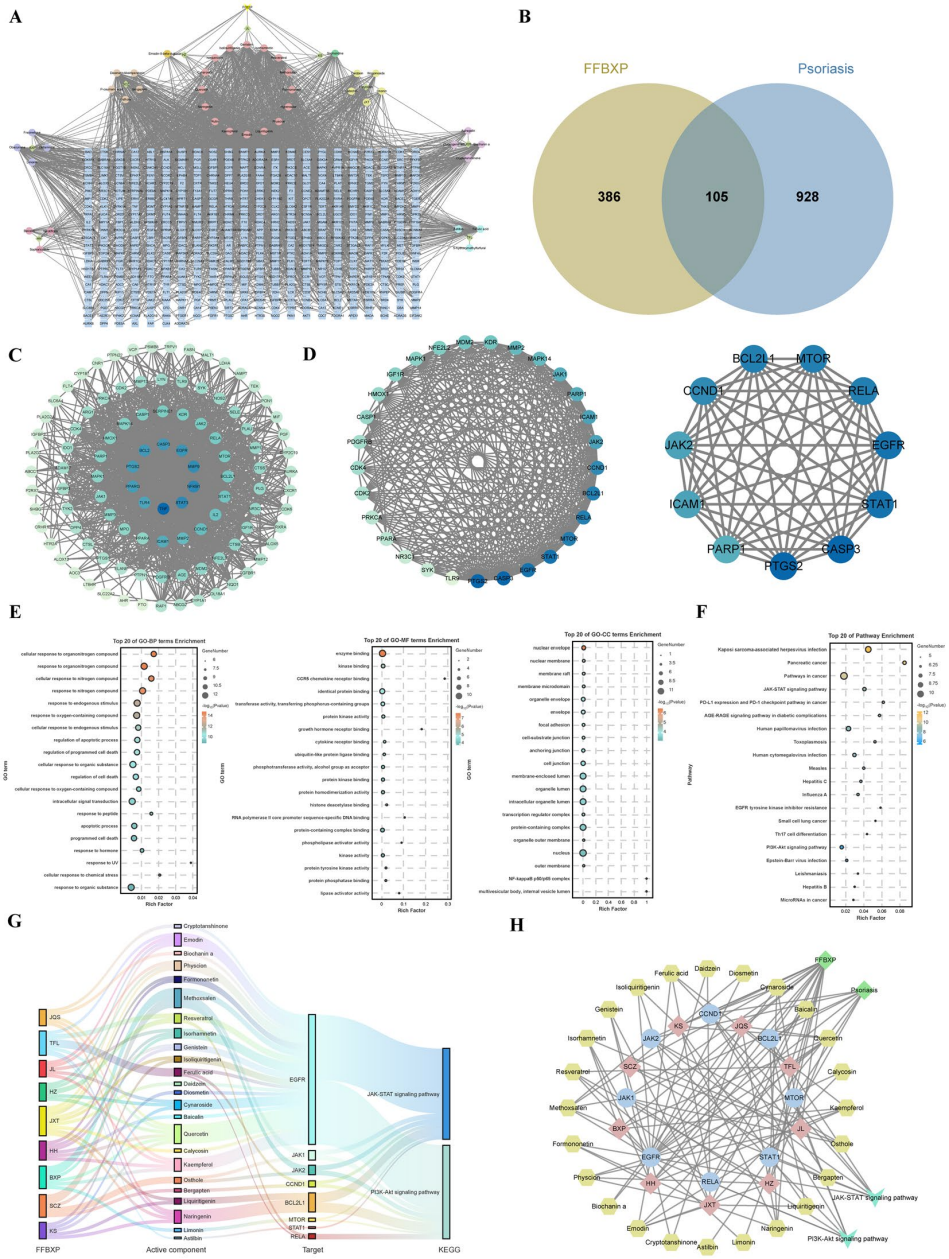
Network pharmacology analysis of FFBXP against psoriasis. **A** FFBXP active compound -target map. **B** Venn plot of FFBXP and psoriasis intersection targets. **C** PPI network. **D** Selection of core targets. **E** GO enrichment analysis. **F** KEGG enrichment analysis. **G** FFBXP active component-target-pathway sankey diagram. **H** FFBXP active component-target-pathway-psoriasis network diagram

Network pharmacology analysis identified 491 potential targets for FFBXP's 42 active compounds. Intersection with psoriasis-related targets (1,033 from GeneCards) yielded 105 overlapping targets (Fig. 3A, B). STRING analysis of the 105 common targets generated an interaction network and visualized via Cytoscape software (Fig. 3C). Cytoscape's MCODE analysis revealed a significant target cluster (score = 20.929) comprising 29 key proteins. Using the CytoNCA plugin, we selected the top 12 highly connected nodes as the core network, including JAK2, JAK1, and STAT1, (Fig. 3D).

GO and KEGG enrichment analyses (Fig. 3E, F) revealed the potential mechanisms of FFBXP in treating psoriasis. GO analysis identified 2,037 significant terms, primarily categorized into biological processes, cellular components, and molecular functions. KEGG analysis identified 102 enriched pathways, among which the JAK-STAT and PI3K-AKT signaling pathways, as well as TH17 cell differentiation, were predicted to be the key pathways mediating FFBXP’s effects. We visualized FFBXP-psoriasis interactions through a sankey diagram (Fig. 3G) and a network (45 nodes/118 edges) of top pathways and targets (Fig. 3H), further clarifying the key compound-target-pathway axis of FFBXP in psoriasis treatment.

### Transcriptomics analysis of FFBXP against psoriasis

**Fig. 4:**
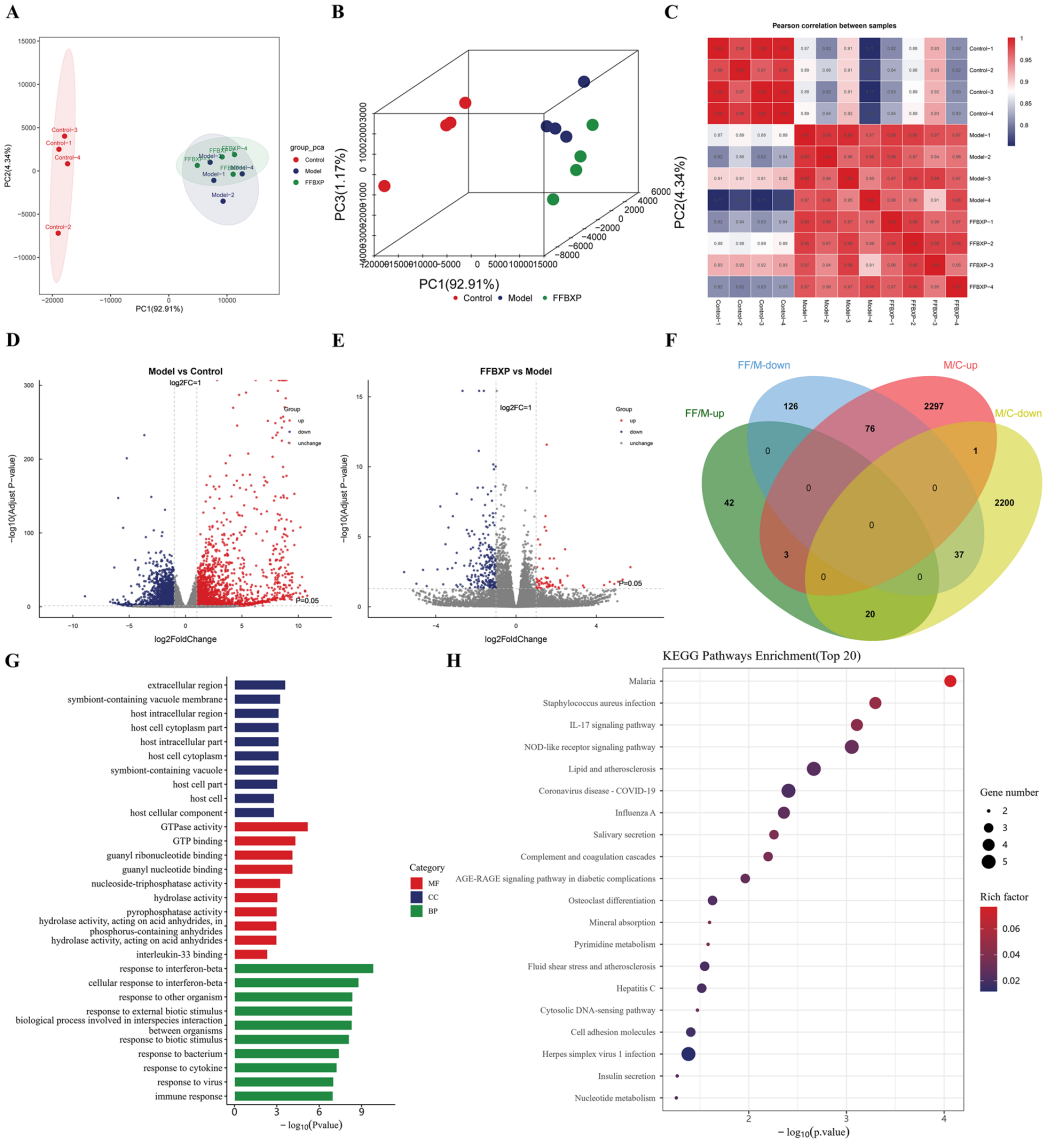
Transcriptomics analysis of FFBXP against psoriasis. **A** PCA score plots-2D. **B** PCA score plots-3D. **C** Heat map of correlation coefficient between samples. **D** Volcanic map of differential gene expression distribution (Model vs Control). **E** Volcanic map of differential gene expression distribution (FFBXP vs Model). **F** Venn maps of upregulated and downregulated genes in different comparison groups. **G** Differential gene GO enrichment bar chart. **H** KEGG enrichment bubble plot of differentially expressed genes

Quality control (QC) and principal component analysis (PCA) demonstrated good experimental stability and clear group separation (Control/Model/FFBXP) with tight intra-group clustering (Fig. 4A, B). Differential expression analysis (thresholds: |log2FC|> 1, Padj < 0.05) identified 4,646 DEGs in Model vs Control (2,383 upregulated, 2,263 downregulated). FFBXP treatment modulated 304 genes (65 upregulated, 239 downregulated) compared to Model. Integrated analysis revealed 96 reversed DEGs (76 previously upregulated genes were downregulated, 20 downregulated genes were upregulated) post-FFBXP intervention (Fig. 4C–G). These reversed genes, particularly those normalized toward Control group levels, represent potential key targets through which FFBXP exerts its anti-psoriatic effects, likely involved in critical inflammatory and proliferative pathways characteristic of psoriasis pathogenesis.

### Network pharmacology and transcriptome combined analysis—molecular docking

**Fig. 5:**
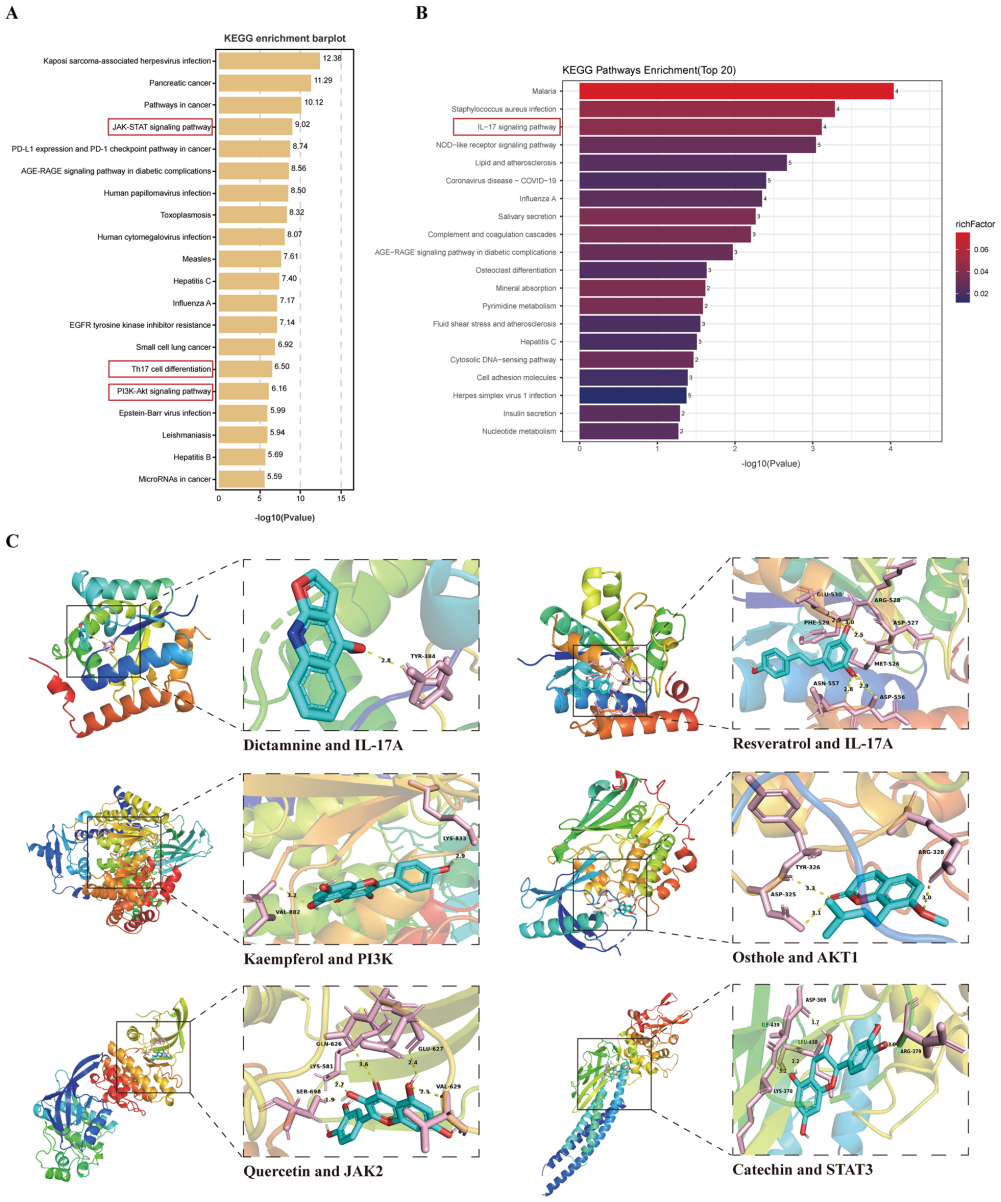
Network pharmacology and transcriptome combined analysis—molecular docking. **A** Network pharmacology-KEGG enrichment bar chart of core targets. **B** Transcriptomics KEGG enrichment bubble plot of differentially expressed genes. **C** The molecular docking diagrams of the six active components of FFBXP and their target molecules

This study combined network pharmacology and transcriptomics to elucidate FFBXP's multi-target anti-psoriatic mechanism. Network analysis identified 12 core targets (e.g.,JAK2, STAT1) and key signaling pathways (PI3K-AKT, JAK2-STAT3) (Fig. 5A). Transcriptomic analysis confirmed FFBXP modulates 96 genes enriched in IL-17 signaling (Fig. 5B). Collectively, these results demonstrate FFBXP's synergistic regulation of PI3K-AKT, JAK2-STAT3, and IL-17 pathways, providing mechanistic insights into its therapeutic effects. Molecular docking simulations revealed six key FFBXP components (Dictamnine, Resveratrol, Quercetin, Catechin, Kaempferol, Osthole) effectively bind to IL-17A, PI3K, AKT, JAK2, and STAT3 proteins (average binding energy: −7.9 kcal/mol), demonstrating stable hydrogen bond interactions (< −5 kcal/mol) (Fig. 5C) (Supplementary Materials 3). To validate the results of molecular docking predictions, we conducted SPR analysis. The results showed that, Quercetin bound strongly to JAK2 (KD = 1.80 μM) with a slow dissociation rate, indicating stable complex formation. Kaempferol exhibited moderate binding to PIK3CA (KD = 3.43 μM) with a fast association rate, while Osthole showed moderate affinity for AKT1 (KD = 5.31 μM). Resveratrol displayed detectable but weaker binding to IL-17A (KD = 217 μM), suggesting possible indirect mechanisms(Fig.S2). These findings indicate FFBXP's multi-component synergy modulates psoriasis-associated pathways via potent target engagement.

### FFBXP ameliorated psoriasis mice through multi-target and multi signaling pathways

**Fig. 6:**
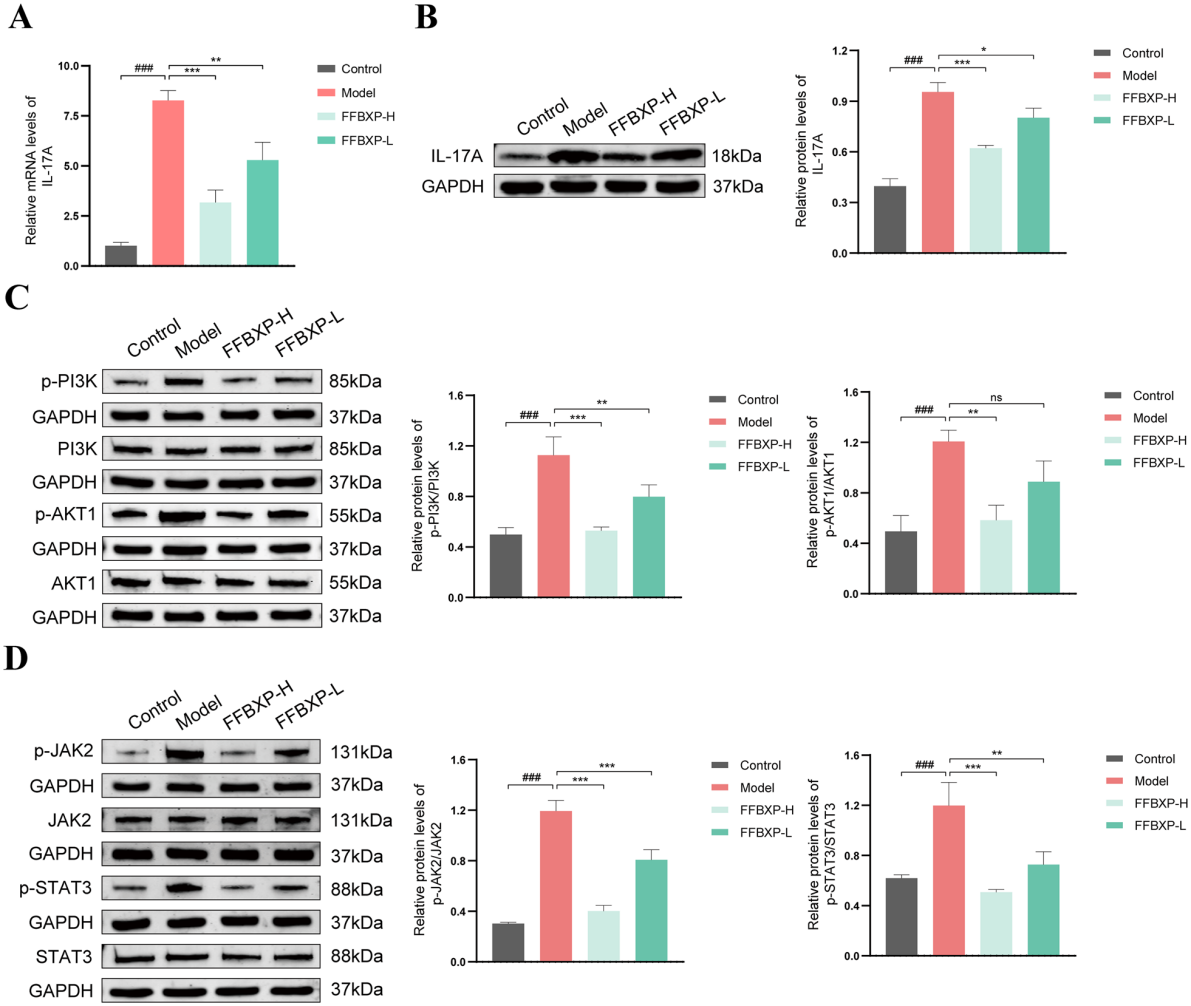
FFBXP ameliorated psoriasis mice through multi-target and multi signaling pathways. **A** The mRNA expression of IL-17A in the skin tissues of each group of mice (*n* = 3). **B** The expression of IL-17A protein in the skin tissues of each group of mice (*n* = 3). **C** The expression of p-PI3K, PI3K, AKT1, and p-AKT1 proteins in the skin tissues of each group of mice (*n* = 3). **D** The expression of p-JAK2, JAK2, STAT3, and p-STAT3 proteins in the skin tissues of each group of mice (*n* = 3). ^###^*p* < 0.001 vs. Control; ^*^p < 0.05; ^**^p < 0.01; ^***^p < 0.001 vs. Model

Experimental validation via qRT-PCR and Western blot confirmed FFBXP's dose-dependent inhibition of psoriasis-associated signaling pathways. Compared with controls, the model group exhibited elevated IL-17A expression (mRNA/protein) (Fig. 6A, B) and increased phosphorylation of p-PI3K, p-AKT1 p-JAK2, and p-STAT3 proteins. FFBXP treatment significantly reduced IL-17A levels and suppressed pathway activation: (1) all doses inhibited p-PI3K, whereas only high-dose inhibited p-AKT1; (2) JAK2-STAT3 was decreased n a dose-dependent manner (Fig. 6C, D). Total protein levels remained unchanged, demonstrating FFBXP specifically targets pathway activation rather than expression. These results confirm that FFBXP alleviates psoriasis by regulating PI3K-AKT/JAK2-STAT3 signaling via IL-17A.

### Evaluation of efficacy of FFBXP in TNF-a induced HaCaT cells

**Fig. 7:**
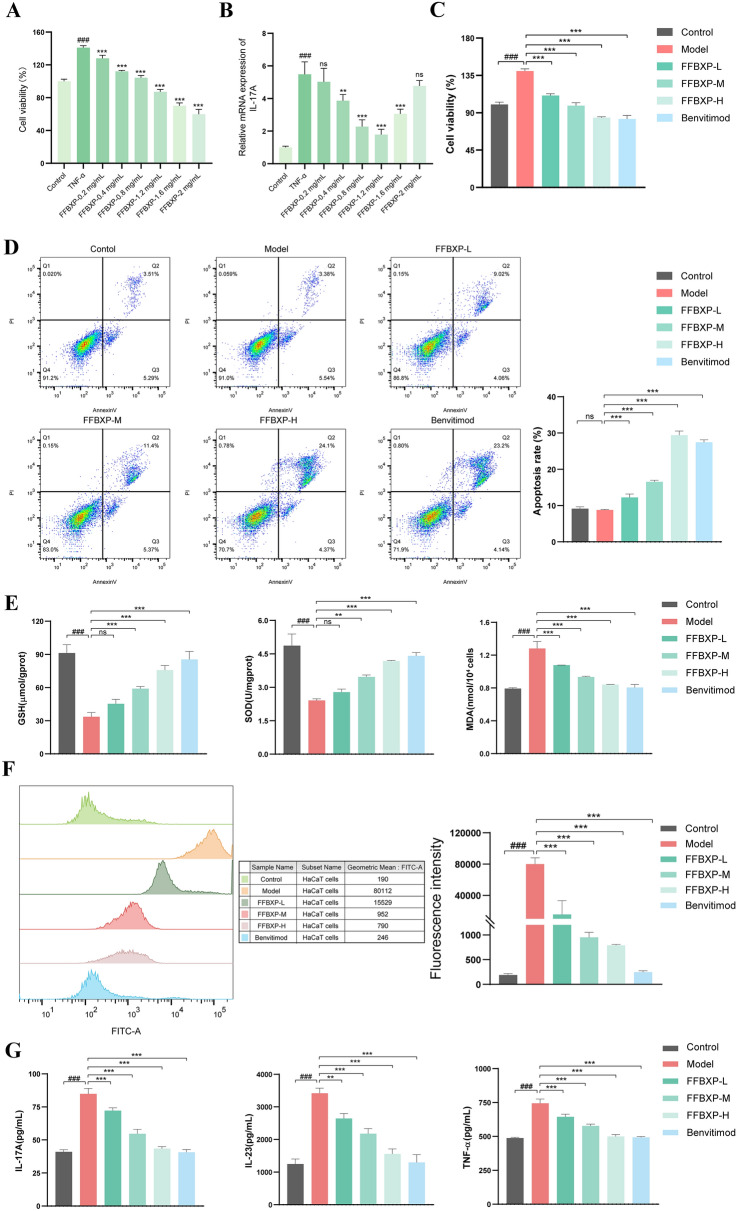
Evaluation of efficacy of FFBXP in TNF-a induced HaCaT cells. **A** The effects of different concentrations of FFBXP on the viability of TNF-α-induced HaCaT cells (*n* = 5). **B** The effects of different concentrations of FFBXP on the mRNA of IL-17A in TNF-α-induced HaCaT cells (*n* = 3). **C** The effects of FFBXP on the proliferation of HaCaT cells (*n* = 5). **D** The effects of FFBXP on the apoptosis of HaCaT cells (*n* = 3). **E** The levels of GSH, SOD, and MDA in each group of HaCaT cells (*n* = 3). **F** The levels of reactive ROS in each group of HaCaT cells (*n* = 3). **G** The levels of IL-17A, IL-23, and TNF-α in the supernatants of HaCaT cells in each group (*n* = 3). ^###^*p* < 0.001 vs. Control; ^**^p < 0.01; ^***^p < 0.001 vs. Model

Using a TNF-α-induced HaCaT hyperproliferation model (10 ng/mL, 24 h), FFBXP (0.2, 0.4, 0.8, 1.2, 1.6, 2 mg/mL) demonstrated dose-dependent inhibition of keratinocyte viability after 48 h treatment (Fig. 7A). While TNF-α stimulated HaCaT proliferation (psoriasis-like state), FFBXP effectively counteracted this abnormal growth, confirming its anti-proliferative potential in psoriatic pathology.

In TNF-α-stimulated HaCaT cells (psoriasis model), FFBXP exhibited concentration-dependent therapeutic effects at 0.4–1.2 mg/mL. qPCR analysis demonstrated significant reduction of pathogenic IL-17A mRNA overexpression (Fig. 7B, C), confirming anti-inflammatory activity. Flow cytometry revealed FFBXP's pro-apoptotic effect, with apoptosis rates increasing progressively across FFBXP-L (0.4 mg/mL), FFBXP-M (0.8 mg/mL), and FFBXP-H (1.2 mg/mL) doses. Notably, the FFBXP-H group induced apoptosis equivalent to the benvitimod positive control (Fig. 7D). These complementary mechanisms—suppressing the psoriasis-associated IL-17A cytokine while restoring normal keratinocyte apoptosis—demonstrate FFBXP's potential to concurrently target both the inflammatory and hyperproliferative aspects of psoriatic pathogenesis at the cellular level.

FFBXP demonstrated comprehensive antioxidant and anti-inflammatory effects in TNF-α-stimulated HaCaT cells. The model group exhibited oxidative stress imbalance (reduced GSH/SOD, increased MDA/ROS), which FFBXP dose-dependently reversed—medium/high doses restored GSH/SOD, while all doses reduced MDA and ROS (Fig. 7E, F). ELISA revealed FFBXP concentration-dependently suppressed TNF-α-induced secretion of IL-17A, IL-23, and TNF-α, with FFBXP-H (1.2 mg/mL) matching benvitimod's efficacy (Fig. 7G). These results demonstrate FFBXP simultaneously alleviates oxidative stress (via ROS scavenging and antioxidant restoration) and inflammation (via cytokine suppression), addressing key pathological features of psoriasis. The coordinated improvement of both oxidative and inflammatory markers suggests FFBXP's multi-target therapeutic potential for psoriasis treatment.

### FFBXP ameliorated TNF-a induced HaCaT cells through multi-target and multi signaling pathways

**Fig. 8:**
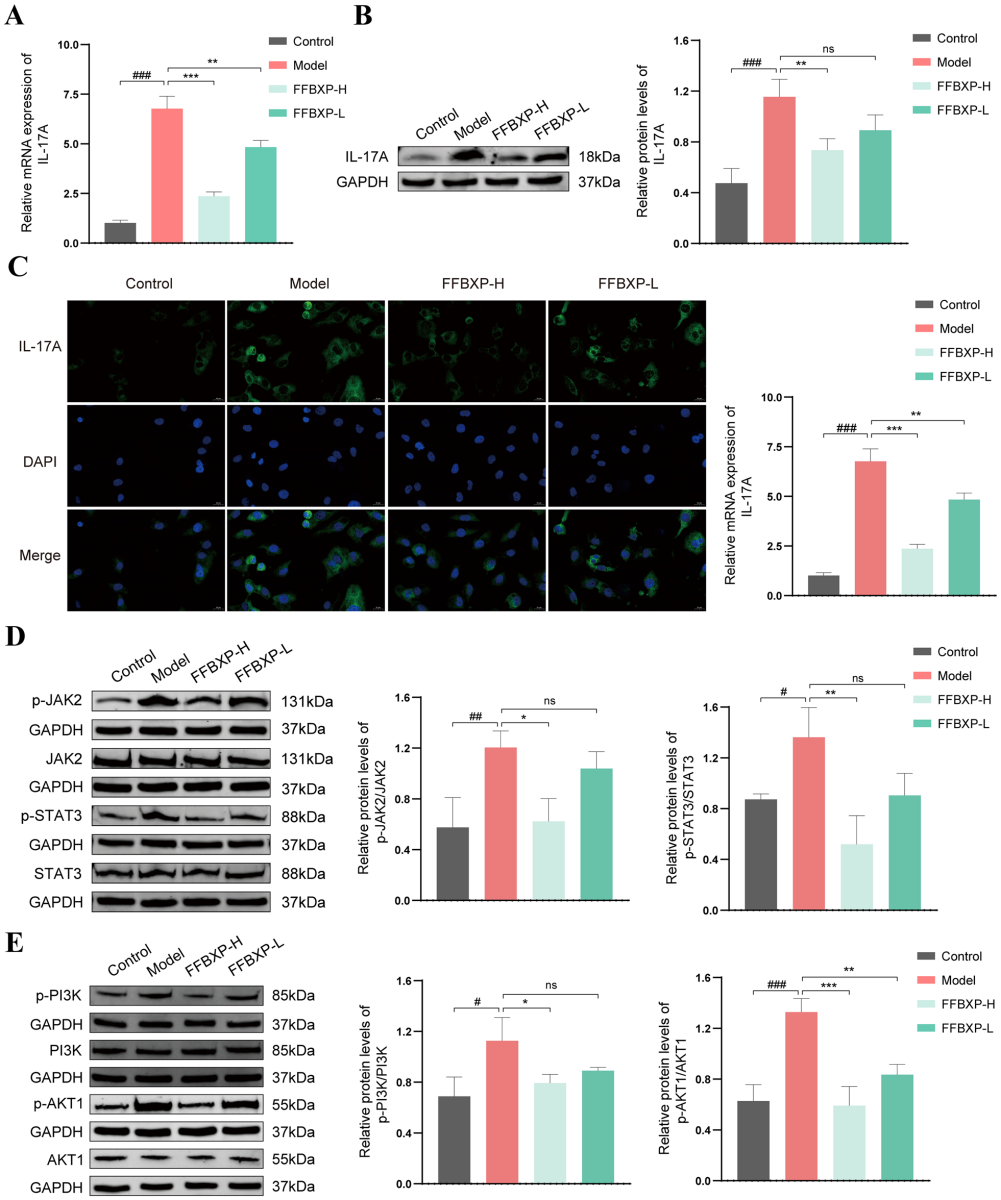
FFBXP ameliorated TNF-a induced HaCaT cells through multi-target and multi signaling pathways. **A** The mRNA expression of IL-17A in each group of HaCaT cells (*n* = 3). **B** The expression of IL-17A protein in each group of HaCaT cells (*n* = 3). **C** The immunofluorescence expression of IL-17A in each group of HaCaT cells (*n* = 3). **D** The expression of p-PI3K, PI3K, AKT1, and p-AKT1 proteins in each group of HaCaTcells (*n* = 3). **E** The expression of p-JAK2, JAK2, STAT3, and p-STAT3 proteins in each group of HaCaT cells (*n* = 3). ^#^*p* < 0.05; ^##^*p* < 0.01; ^###^*p* < 0.001 vs. Control; ^*^p < 0.05; ^**^p < 0.01; ^***^p < 0.001 vs. Model

qRT-PCR and WB analyses revealed that FFBXP dose-dependently suppressed TNF-α-induced IL-17A mRNA and protein overexpression in HaCaT cells (Fig. 8A, B). Immunofluorescence confirmed significantly reduced IL-17A intensity post-FFBXP treatment (Fig. 8C), demonstrating potent inhibition of this key psoriatic cytokine. WB analysis revealed that FFBXP significantly inhibited the phosphorylation of p-PI3K, p-AKT1, p-JAK2, and p-STAT3 proteins in HaCaT cells (Fig. 8D, E), while total protein levels remained unchanged. The results confirm that FFBXP exerts its anti-psoriatic effects via a targeted mechanism, specifically suppressing the abnormal activation of key signaling pathways while preserving normal protein expression levels.

## Discussion

This study represents the first integrated approach combining network pharmacology prediction, transcriptomics screening and experimental validation, establishing a three-dimensional research strategy of computational prediction-omics screening-experimental confirmation to systematically elucidate the mechanism of action of FFBXP. Network pharmacology overcomes reductionism limitations by integrating systems biology and bioinformatics to construct comprehensive "component-target-pathway" networks, enabling systematic analysis of TCM's holistic therapeutic effects [32]. Similarly transcriptomics reveals genome-wide gene expression changes following drug intervention [33]. Their integrated analysis predicted that FFBXP might regulate the PI3K-AKT and JAK2-STAT3 pathways via IL-17A. This prediction was systematically validated through animal experiments, cellular assays, and SPR technology, forming a complete evidence chain from in silico prediction to experimental confirmation. This multidimensional strategy provides a methodological paradigm for investigating the mechanisms of complex TCM formulations.

We established a psoriasis mouse model using imiquimod cream, which was confirmed by characteristic scales, erythema and elevated PASI scores. After 7-day FFBXP treatment, skin lesions significantly improved with reduced erythema, scaling and epidermal thickening. Histology showed normalized keratinocyte proliferation and decreased inflammation. Psoriasis is frequently accompanied by localized inflammation, where the secretion of immune cells and pro-inflammatory cytokines further exacerbates the inflammatory cascade [34]. Patients with psoriasis exhibit impaired antioxidant defense mechanisms, leading to increased oxidative stress and subsequent skin damage [35]. Excessive ROS and MDA cause cell damage, activating immune cells and inflammation, while antioxidants (GSH, SOD) counteract oxidative stress and protect cells [36]. FFBXP treatment effectively alleviated psoriasis skin damage by reducing inflammatory cytokines, enhancing antioxidant defenses (GSH/SOD), and decreasing oxidative stress (MDA), demonstrating its dual anti-inflammatory and antioxidant mechanisms (Fig. 2D).

TNF-α, a key pro-inflammatory factor in psoriasis, induces HaCaT cells (human immortalized keratinocytes) to undergo abnormal proliferation and inflammatory responses characteristic of the disease [37, 38]. Consequently, this study used a TNF-α-induced HaCaT cell model to simulate the pathological state of psoriasis. The results showed that FFBXP dose-dependently inhibited HaCaT cell proliferation, reduced IL-17A expression, and promoted cell apoptosis (Fig. 7). Additionally, FFBXP decreased the release of inflammatory factors (IL-17A, IL-23, TNF-α). It also alleviated oxidative stress by reducing ROS and MDA while restoring GSH and SOD levels (Fig. 7E–G). These results confirm that FFBXP ameliorates psoriasis-like keratinocyte abnormalities through anti-inflammatory, antioxidant, and pro-apoptotic effects.

IL-17A is a key inflammatory factor; the PI3K-AKT pathway is associated with keratinocyte hyperproliferation, and the JAK2-STAT3 pathway mediates inflammatory responses and abnormal keratinocyte proliferation [39–42]. These three signaling pathways jointly contribute to psoriasis pathogenesis and may interact to modulate disease progression. Our research confirms that IL-17A, PI3K-AKT, and JAK2-STAT3 constitute key pathways targeted by FFBXP. In IMQ-induced psoriatic mice and TNF-α-stimulated HaCaT cells, FFBXP significantly reduced IL-17A expression and inhibited the phosphorylation of PI3K, AKT, JAK2, and STAT3 (Figs. 6 and 8). Notably, IL-17A can activate JAK2-STAT3 signaling, forming a positive feedback loop that exacerbates psoriasis progression [43]. FFBXP's ability to disrupt this loop likely contributes to its therapeutic efficacy. Furthermore, SPR analysis provided direct evidence that key FFBXP components bind to these pathway proteins: quercetin bound to JAK2 with high affinity (KD = 1.80 μM), kaempferol bound to PIK3CA (KD = 3.43 μM), osthole bound to AKT1 (KD = 5.31 μM), and resveratrol bound to IL-17A (KD = 217 μM) (Fig. S2). These findings demonstrate that multiple components of FFBXP collectively target the IL-17A/PI3K-AKT/JAK2-STAT3 pathway, exemplifying the synergistic multi-target action characteristic of TCM formulations.

Although this study elucidated FFBXP's anti-psoriasis mechanism, three limitations should be acknowledged. First, target validation remains incomplete; many potential component-target interactions require further validation. Second, the disease models have limited clinical relevance, as the IMQ-induced model mimics acute inflammation and HaCaT cells differ from primary keratinocytes. Third, regarding administration, the stability of FFBXP's active components during wet compresses was not assessed, and this method differs from clinical application of creams or ointments.

Future studies should address these limitations by: (1) validating more component-target interactions using techniques such as CETSA and MST; (2) employing clinically relevant models like humanized mice or skin organoids; and (3) optimizing FFBXP's formulation with pharmacokinetic studies to facilitate clinical translation.

## Conclusion

**Fig. 9:**
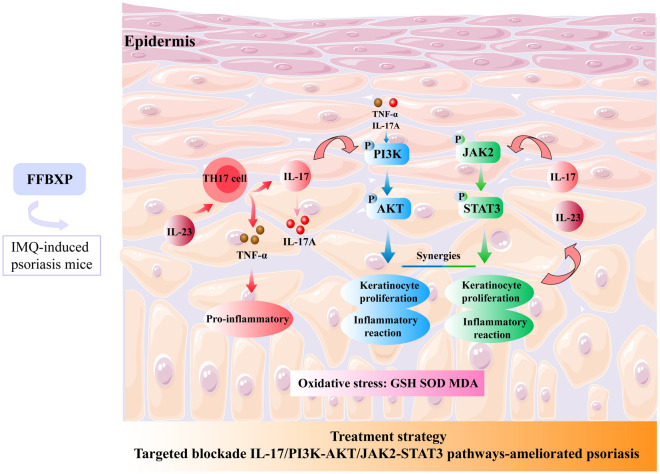
The molecular mechanism of FFBXP treatment for IMQ-induced psoriasis

Integrating network pharmacology, transcriptomics, molecular docking, and SPR analysis, we demonstrated that FFBXP mitigates psoriasis-like inflammation by modulating the IL-17A-mediated PI3K-AKT and JAK2-STAT3 signaling pathway. In both murine models and HaCaT cell cultures, FFBXP attenuated skin lesions, suppressed epidermal keratinocyte hyperproliferation, and alleviated inflammatory responses and oxidative stress through regulation of this pathway (Fig. 9). These findings validate FFBXP as a promising local treatment for psoriasis, providing a scientific rationale for its clinical translation.

## Supplementary Information

Supplementary material 1.

Supplementary material 2.

Supplementary material 3.

Supplementary material 4.

Supplementary material 5.

Supplementary material 6.

## Data Availability

Data presented in the main manuscript or Supplementary material. Additional data which were not included will be provided on reasonable request.

## Abbreviations

FFBXP: Compound Baixianpi Formula
PASI: Psoriasis area and severity index
qRT-PCR: Quantitative real-time polymerase chain reaction
UHPLC-Q-Orbitrap HRMS: Ultra-high-performance liquid chromatography-Q-Orbitrap-high-resolution mass spectrometry
TIC: Total ion chromatogram
HaCaTcells: Human immortalized epidermal cells
H&E: Hematoxylin & Eosin
CCK-8: Cell counting kit-8
ELISA: Enzyme-linked immunosorbent assay
IL-17A: Interleukin-17A
IL-23: Interleukin-23
TNF-α: Tumor necrosis factor alpha
MDA: Malonaldehyde
SOD: Superoxide dismutase
GSH-Px: Glutathione peroxidase
