# Supplementary material for: Integrating network pharmacology, transcriptomics, and experimental validation: Compound Baixianpi Formula targets IL-17A to inhibit dual PI3K-AKT/JAK2-STAT3 pathways for psoriasis improvement
Source: Chin Med. 2026 May 22;21:141. doi: 10.1186/s13020-026-01386-0 (PMC13196228; doi:10.1186/s13020-026-01386-0)
Supplement: Supplementary file 1 — Supplementary material 1. [file 13020_2026_1386_MOESM1_ESM.docx]

**A**


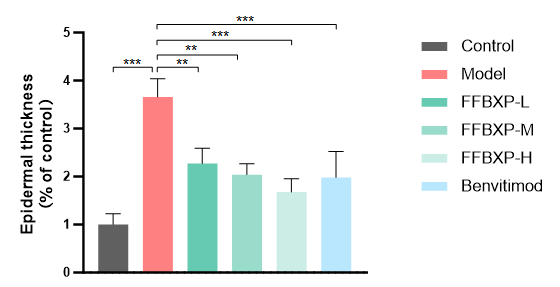


**B**


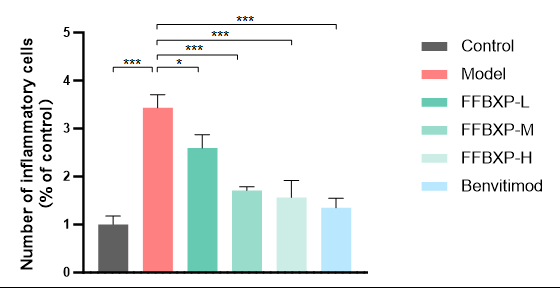


**Fig. S1.** Quantitative analysis of histopathological changes. (A) Epidermal thickness. (B) Inflammatory cell counts. Data are mean ± SD (n=3). *p < 0.05，**p < 0.01，***p < 0.001 vs. Model.
