# Supplementary material for: Integrating network pharmacology, transcriptomics, and experimental validation: Compound Baixianpi Formula targets IL-17A to inhibit dual PI3K-AKT/JAK2-STAT3 pathways for psoriasis improvement
Source: Chin Med. 2026 May 22;21:141. doi: 10.1186/s13020-026-01386-0 (PMC13196228; doi:10.1186/s13020-026-01386-0)
Supplement: Supplementary file 2 — Supplementary material 2. [file 13020_2026_1386_MOESM2_ESM.docx]

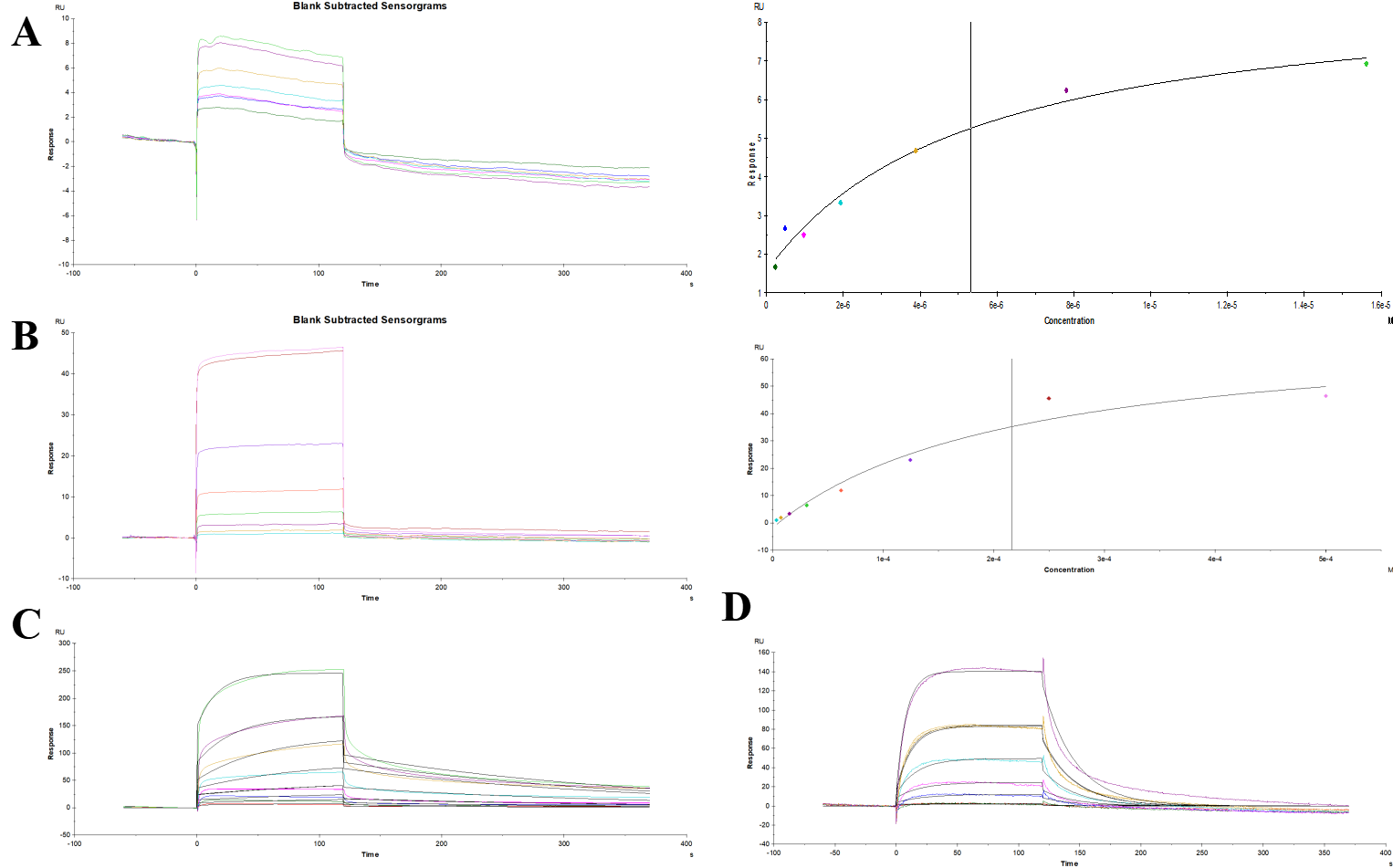


**Fig. S2.** (A) Sensorgram showing the interaction of AKT1 protein with Osthole, yielding Kd = 5.31 μM. (B) Sensorgram showing the interaction of IL17A protein with Resveratrol, yielding Kd = 217 μM. (C) Sensorgram showing the interaction of JAK2 protein with Quercetin, yielding Kd = 1.80 μM. (D) Sensorgram showing the interaction of PIK3CA protein with Kaempferol, yielding Kd = 3.43 μM.
