# Supplementary material for: Integrating network pharmacology, transcriptomics, and experimental validation: Compound Baixianpi Formula targets IL-17A to inhibit dual PI3K-AKT/JAK2-STAT3 pathways for psoriasis improvement
Source: Chin Med. 2026 May 22;21:141. doi: 10.1186/s13020-026-01386-0 (PMC13196228; doi:10.1186/s13020-026-01386-0)
Supplement: Supplementary file 3 — Supplementary material 3. [file 13020_2026_1386_MOESM3_ESM.docx]

**Supplementary materials 1**

PASI scoring criteria for the back of mice

| Score | erythema | Scale | Infiltration |
| --- | --- | --- | --- |
| 0 | No erythema was observed | No scales were seen on the surface | The lesions are level with the normal skin |
| 1 | light red | The surface of a few lesions is covered with scales, mainly fine scales | The lesions are slightly higher than normal skin Surface of the skin |
| 2 | bright red | Most of the lesions are covered with scales, which are sheet-like | Moderate elevation, with circular or sloping borders of the plaque |
| 3 | dark red | Almost all lesions are covered with scales that are thick and layered | The lesions are relatively thick and prominent |
| 4 | Extremely deep red | All lesions are covered with thick scales and layered | The lesions are highly thickened and very prominent |
