# Supplementary material for: Integrating network pharmacology, transcriptomics, and experimental validation: Compound Baixianpi Formula targets IL-17A to inhibit dual PI3K-AKT/JAK2-STAT3 pathways for psoriasis improvement
Source: Chin Med. 2026 May 22;21:141. doi: 10.1186/s13020-026-01386-0 (PMC13196228; doi:10.1186/s13020-026-01386-0)
Supplement: Supplementary file 4 — Supplementary material 4. [file 13020_2026_1386_MOESM4_ESM.docx]

**Supplementary materials 2**

| **Gene** | **Primer** |
| --- | --- |
| mouse-β-GAPDH | Forward 5'-AGGTCGGTGTGAACGGATTTG-3'  Reverse 5'-GGGGTCGTTGATGGCAACA-3' |
| mouse-IL-17A | Forward 5'-TCAGCGTGTCCAAACACTGAG-3'  Reverse 5'-CGCCAAGGGAGTTAAAGACTT-3' |
| human-GAPDH | Forward 5'-GGAGCGAGATCCCTCCAAAAT-3'  Reverse 5'-GGCTGTTGTCATACTTCTCATGG-3' |
| human-IL-17A | Forward 5'-TCCCACGAAATCCAGGATGC-3'  Reverse 5'-GGATGTTCAGGTTGACCATCAC-3' |
