# Supplementary material for: Integrating network pharmacology, transcriptomics, and experimental validation: Compound Baixianpi Formula targets IL-17A to inhibit dual PI3K-AKT/JAK2-STAT3 pathways for psoriasis improvement
Source: Chin Med. 2026 May 22;21:141. doi: 10.1186/s13020-026-01386-0 (PMC13196228; doi:10.1186/s13020-026-01386-0)
Supplement: Supplementary file 5 — Supplementary material 5. [file 13020_2026_1386_MOESM5_ESM.docx]

**Supplementary materials 3**

**FFBXP molecular docking data**

| **Gene ID** | **PBD ID** | **Compounds** | **Estimated ΔG (kcal/mol)** |
| --- | --- | --- | --- |
| IL-17 | 4nux | Dictamnine | -6.3 |
| IL-17 | 4nux | Resveratrol | -7.3 |
| JAK2 | 7f7w | Quercetin | -9.3 |
| STAT3 | 6njs | Catechin | -7.4 |
| PI3K | 5jhb | Kaempferol | -8.2 |
| AKT1 | 6hhg | Osthole | -8.7 |
