# Supplementary figures and images for: Integrating network pharmacology, transcriptomics, and experimental validation: Compound Baixianpi Formula targets IL-17A to inhibit dual PI3K-AKT/JAK2-STAT3 pathways for psoriasis improvement
Source: Chin Med. 2026 May 22;21:141. doi: 10.1186/s13020-026-01386-0 (PMC13196228; doi:10.1186/s13020-026-01386-0)

**
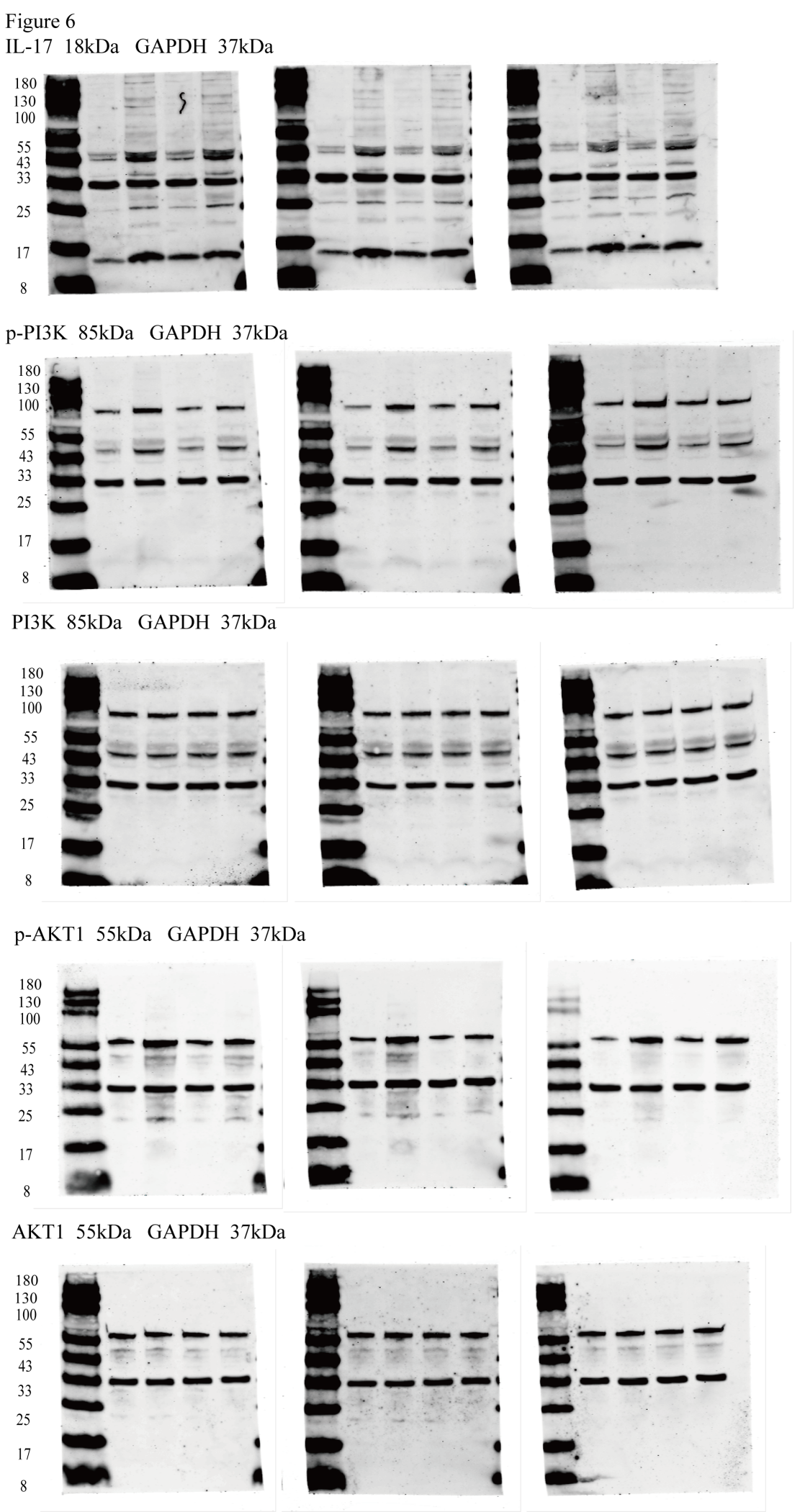
**

**
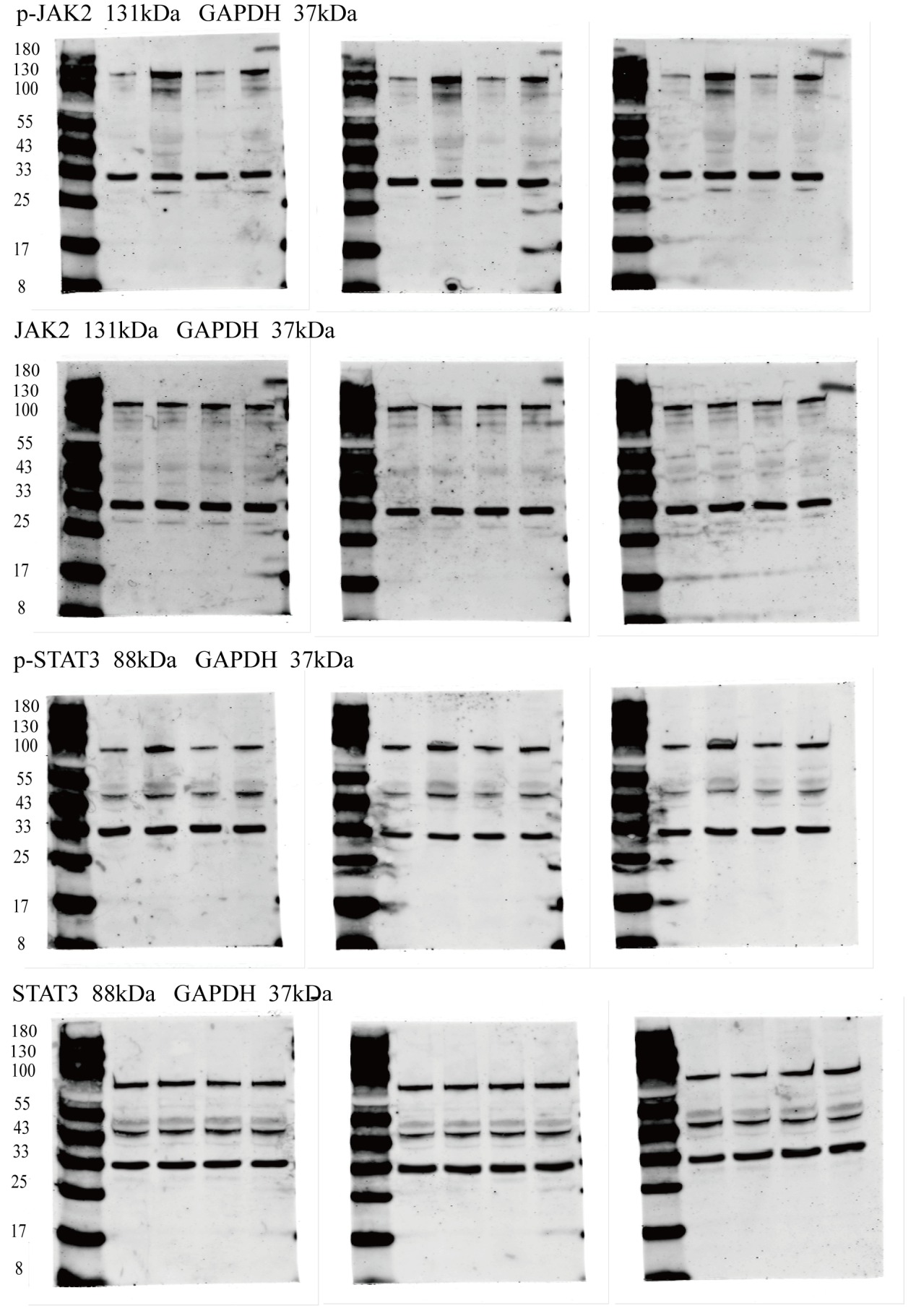
**

**
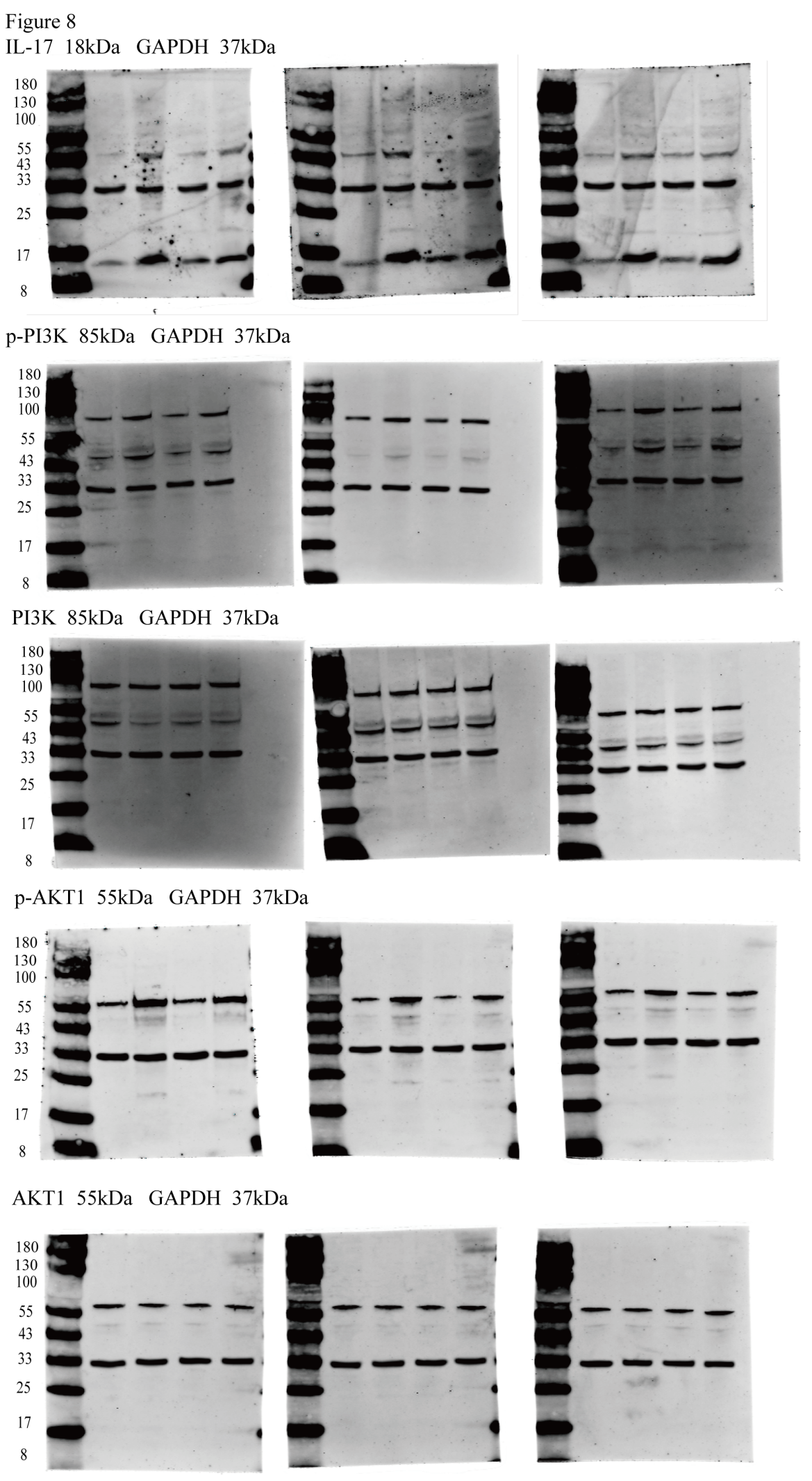
**

**
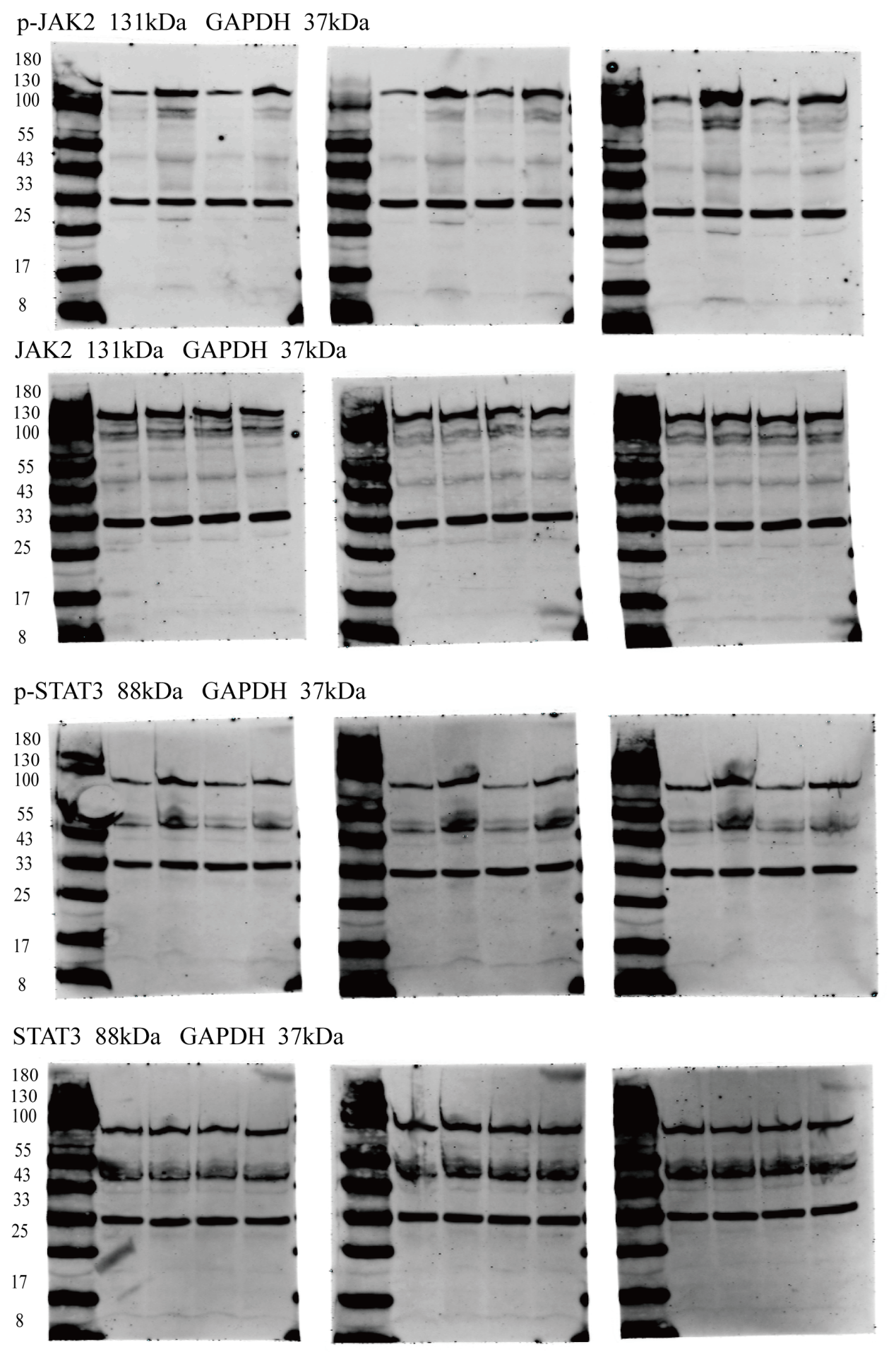
**

Supplement: Supplementary file 6 — Supplementary material 6. [file 13020_2026_1386_MOESM6_ESM.docx]
